# A method for the growth of uniform silica shells on different size and morphology upconversion nanoparticles†‡

**DOI:** 10.1039/d0na00858c

**Published:** 2021-05-10

**Authors:** Elena Ureña-Horno, Maria-Eleni Kyriazi, Antonios G. Kanaras

**Affiliations:** Physics and Astronomy, Faculty of Physical Sciences and Engineering, University of Southampton Southampton SO171BJ UK a.kanaras@soton.ac.uk; Institute for Life Science, University of Southampton Southampton SO171BJ UK

## Abstract

Lanthanide-doped upconversion nanoparticles have emerged as attractive candidates for biomedical applications. This is due to their excitation and emission wavelengths, which lay the foundation for deeper penetration depth into biological tissue, higher resolution due to reduced scattering and improved imaging contrast as a result of a decrease in autofluorescence background. Usually, their encapsulation within a biocompatible silica shell is a requirement for their dispersion within complex media or for further functionalization of the upconversion nanoparticle surface. However, the creation of a silica shell around upconversion nanoparticles can be often challenging, many times resulting in partial silica coating or nanoparticle aggregation, as well as the production of a large number of silica particles as a side product. In this work we demonstrate a method to accurately predict the experimental conditions required to form a high yield of silica-coated upconversion nanoparticles, regardless of their shape and size.

## Abstract 2

Silica shell growth on lanthanide-doped upconversion nanoparticles.

## Graphical abstract

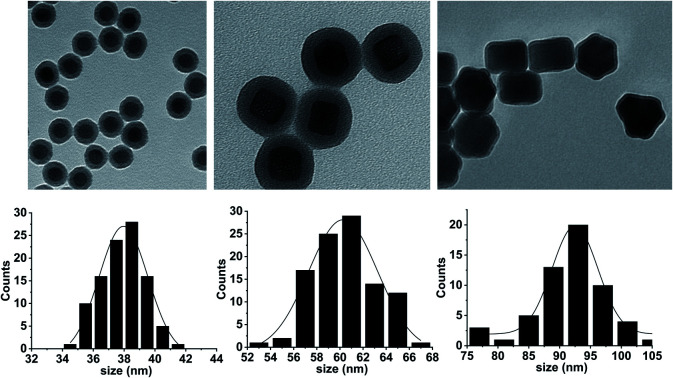

## Introduction

Rare earth doped glass nanoparticles such as NaYF_4_: Yb^3+^, Er^3+^, also termed upconversion nanoparticles (UCNPs), have recently attracted scientific interest due to their unique fluorescent properties.^1^ These nanoparticles absorb two or more near-IR photons and emit at the visible area as a result of 4f electronic transitions. They possess excellent chemical stability, large stokes shifts, narrow bandwidth, lack of photobleaching and photoblinking, and reduced toxicity.^2,3^ Although UCNPs do not have a large quantum yield, due to the multiple electronic transitions and relevant losses, their unique ability to absorb light in the near IR (typically 980 nm) and emit at the UV-vis make them relevant for applications within the energy and biomedical fields, where they demonstrate a high signal to noise ratio and lack background auto-fluorescence.^2–8^

UCNPs are usually synthesized in the organic phase following a co-precipitation method, which results in nanoparticles coated with oleylamine (OM) or oleic acid (OA) ligands.^9^ Post ligand exchange or surface modification is vital to transfer the nanoparticles to the water phase, and it is required for their use in biomedical applications. One method currently employed to achieve water solubility is their encapsulation within a silica shell. Encapsulation of UCNPs within a silica shell further equips nanoparticles with biocompatibility, chemical stability and the ability to further conjugate their surface with alternative functional groups such as synthetic oligonucleotides.^9–13^

To date, the reverse microemulsion approach is the most common procedure employed for silica coating of hydrophobic UCNPs^7,14–16^ and relies on the use of an amphiphilic surfactant such as polyoxyethylene (5) nonyl – phenylether (trade name Igepal CO – 520), ammonia and tetraethyl orthosilicate (TEOS).^17–21^ The reaction involves several steps as shown in Scheme S1.† First the surfactant (Igepal CO-520) is introduced to the organic solvent and due to its amphiphilic nature, it forms micelles with the hydrophobic groups extended to the outside and the hydrophilic groups inwards. When OA coated nanoparticles are added to the solution, a ligand exchange between OA and Igepal CO-520 occurs on the nanoparticle surface with part of the surfactant still in the micelle form. Ammonia is then added, and the micelle size is enlarged to form the reverse microemulsion system where the size of the resulting micelle can be controlled by adjusting the ratio of ammonia to Igepal CO-520. Subsequently TEOS is added to the solution and is hydrolysed. Hydrolysed TEOS then replaces the Igepal CO-520, which is chemically absorbed on the nanoparticle surface. Then nanoparticles are transferred into the micelle where the hydrolysed TEOS on the nanoparticle surface can undergo a condensation process and form a silica shell.^22^

Although the mechanism of the microemulsion method is well understood there is a lack of adequate control of the reaction, which results in the formation of silica nanoparticles (SiO_2_ NPs) as a side product.^16,22,23^ These particles cannot be easily separated from the silica-coated UCNPs, which presents an obstacle when used for further applications. Li *et al.* proposed that matching the number of iron oxide nanoparticles with the number of aqueous micelles is essential to obtain an adequate yield of silica-coated nanoparticles. However, when keeping all experimental parameters constant an increase in the iron oxide nanoparticle size led to the appearance of a large number of SiO_2_ NPs as a side product, which was observed through transmission electron microscopy (TEM).^22^ Thus, they suggested that a successful encapsulation was strongly dependant not only on the molar ratio of Igepal CO-520 to ammonia, which determined the micelle size but also on the size of the hydrophobic iron oxide nanoparticles.^22–25^

Another drawback to the reverse microemulsion method is the encapsulation of multiple nanoparticles within the same silica shell, which leads to encapsulated nanoparticle agglomerates or aggregates.^20,22,26^ Regulating the amount of TEOS and the initial concentration of nanoparticles has been suggested as crucial parameters to avoid the encapsulation of multiple nanoparticles in silica. For example, Yang and co-workers found that decreasing the amount of TEOS lead to the encapsulation of multiple QD cores in silica. They hypothesized that a lower concentration of TEOS resulted in less negative silica intermediates. This in turn reduced the repulsive interactions between neighbouring nanoparticles and lead to the encapsulation of several QDs.^27^

The aforementioned drawbacks hinder the production of high yield and high-quality silica-coated nanoparticles. Moreover, the ambiguous coating parameters that cannot be easily adapted to different shape and size nanoparticles limit their broader applicability. Therefore, there is a lack of a general experimental protocol to produce a high yield of silica-coated inorganic nanoparticles that takes into consideration the morphology and size of OA-coated nanoparticles. In this work, we introduce a universal methodology to coat UCNPs with a silica shell based on the nanoparticle surface area. We demonstrate that by tuning simple parameters, different sizes and morphologies of UCNPs can be efficiently encapsulated in a silica shell avoiding the production of SiO_2_ NPs or multiple nanoparticles encapsulated within the same silica shell.

## Results and discussion

### Synthesis and characterization of UCNPs of different size and morphology

**Fig. 1:**
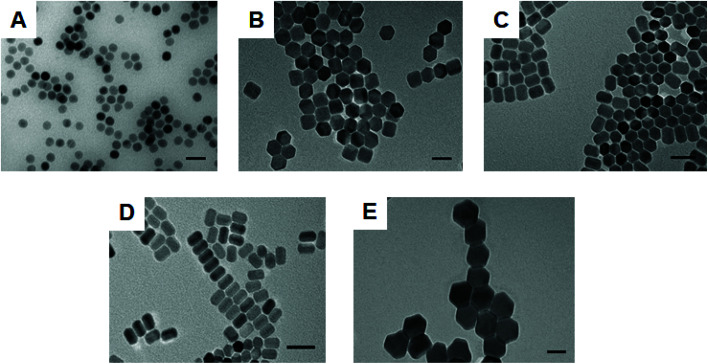
A gallery of various shape and size UCNPs synthesized in-house. Representative TEM images of (A) spherical NaYF_4_: Yb^3+^ (20%), Er^3+^ (2%) UCNPs (sample 1); (B) hexagonal NaYF_4_: Yb^3+^ (20%), Er^3+^ (2%) UCNPs (sample 2); (C) rod-shaped NaYF_4_: Yb^3+^ (20%), Er^3+^ (2%) UCNPs with aspect ratio 1.28 (sample 3); (D) rod-shaped NaYF_4_: Yb^3+^ (20%), Er^3+^ (2%) UCNPs with aspect ratio 1.47 (sample 4); (E) large hexagonal NaY (28%)/Lu (50%) F_4_: Yb^3+^ (20%), Er^3+^ (2%) UCNPs. Scale bar is 50 nm.

A variety of size and shape NaYF_4_: Yb^3+^ (20%), Er^3+^ (2%) UCNPs were synthesized by modifying a previously reported experimental protocol.^28^ The range of nanoparticles included spherical (sample 1) and hexagonal (sample 2) shaped UCNPs as well as nanorods (with an elongated hexagonal structure) of two different size distributions (sample 3 and 4). Furthermore, larger hexagonal UCNPs (sample 5) were prepared by co-doping with 50% lutetium for the synthesis of NaY (28%)/Lu (50%) F_4_: Yb^3+^ (20%), Er^3+^ (2%) UCNPs.^29,31^ All types of synthesized particles were firstly characterized by TEM. Fig. 1 shows TEM images of the UCNPs ranging from spherical (Fig. 1A) to hexagonal (Fig. 1B) and nanorods (Fig. 1C and D). The concentration of OA played a key role in obtaining the different particles. By gradually increasing the OA from 6 to 21 ml respectively, whilst keeping all other parameters constant the preferential growth rate in different directions of the crystal was critically affected (see Table S1† for NP dimensions). Indeed, previous studies have shown that at low ligand concentration preferential nanoparticle growth occurs in the [100] direction as opposed to the [001] direction. However, at higher OA concentrations a growth in the [001] direction occurs faster.^28,32^ Significantly larger hexagonal UCNPs were obtained at a low concentration of OA while doping the glass matrix with 50% lutetium (see Fig. 1E). When Y^3+^ is replaced with Lu^3+^ the electron charge density on the surface of UCNPs decreases. This allows for an increased attraction of F^−^ ions to the surface of UCNPs, which benefits the growth process. Thus, co-doping with lutetium was found to suppress nucleation and resulted in a considerable increase in the size of UCNPs. The final nanoparticle size could be controlled by adjusting the percentage of doping with the largest size nanoparticles being obtained with a 50% lutetium doping.^29^ All types of nanoparticles had a narrow size distribution as shown in the histograms obtained from size analysis of a large number of nanoparticles per sample (see Fig. S1 and S2†).

Nanoparticles were further characterised by X-ray Diffraction (XRD) and Fourier-Transform Infrared Spectroscopy (FTIR). In the respective XRD spectra (see Fig. S3†), each sample presented a hexagonal packing of atoms when compared to the pure phase of hexagonal bulk NaYF_4_ (PDF card no.: 00-016-0334). The FTIR spectrum (see Fig. S4†) of each type of nanoparticle showed two main vibrations at 2923 cm^−1^ and 2858 cm^−1^, which were respectively attributed to the asymmetric and symmetric stretching modes of the methylene (–CH_2_) groups in the long alkyl chain of the OA molecule. Furthermore, for each sample two bands at 1559 cm^−1^ and 1459 cm^−1^ appeared and were attributed to the asymmetric and symmetric stretching vibrations of the carboxylate group [(–COO–)_3_Y^3+^] of the UCNPs, respectively.

### Determination of optimum concentration of UCNPs required for growing a silica shell

As discussed earlier, the synthesis of silica-coated UCNPs is often accompanied by the formation of SiO_2_ NPs as a side product. In a reverse microemulsion system, the ratio between Igepal CO-520 and ammonia is crucial and determines the number and size of aqueous micelles formed.^26^ If the number of UCNPs is equal to the number of the aforementioned micelles, a one to one ratio between the number of UCNPs and micelles is satisfied and would ensure that no SiO_2_ NPs would be formed. The La Mer theory has previously been used to qualitatively describe the optimal reaction conditions to avoid the formation of SiO_2_ NPs.^16^ Initially hydrolysed monomers of TEOS are formed. When the concentration of formed monomers (*C*) exceeds the solubility concentration (*C*_s_), heterogeneous nucleation takes place on the nanoparticle surface. However, when the homogeneous nucleation (*C*_homo_) threshold is surpassed spontaneous aggregation takes place. Therefore, both silica-coated hydrophobic UCNPs and SiO_2_ NPs coexist when the monomer concentration is higher than that of homogeneous nucleation (*C* > *C*_homo_). To avoid the appearance of SiO_2_ NPs the monomer concentration must fulfil the condition *C*_s_ < *C* < *C*_homo_ throughout the reaction and the number of UCNPs must equal the number of aqueous micelles in order to achieve a one-to-one silica coating.^22^ Two calibration experimental methods can be employed to achieve this. The first focuses on calibrating the concentration of the TEOS precursor, Igepal CO-520 and ammonia catalyst in relation to a steady concentration of nanoparticles to suppress the development of secondary nuclei. The second method involves keeping the precursors constant whilst changing the concentration of UCNPs.^22^ In our study the later method was adapted as it involved the variation of a single parameter.

**Fig. 2:**
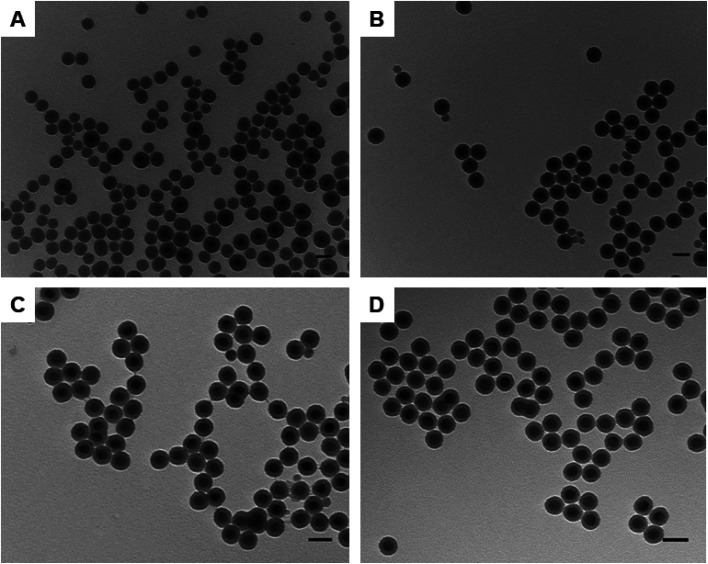
TEM images of silica coated NaYF_4_: Yb^3+^ (20%), Er^3+^ (2%) UCNPs (UCNPs@SiO_2_) synthesised using (A) 0.2 mg ml^−1^ (B) 0.6 mg ml^−1^ (C) 1 mg ml^−1^ and (D) 1.3 mg ml^−1^ of UCNPs while keeping all other parameters (hexane, Igepal Co-520, NH_4_OH and TEOS) constant. Scale bars are 50 nm.

Spherical NaYF_4_: Yb^3+^ (20%), Er^3+^ (2%) UCNPs were initially employed as a template to calibrate the experimental conditions for the silica growth by changing only the amount of UCNPs while keeping all other parameters steady. Fig. 2 shows representative TEM images of silica-coated UCNPs obtained as the amount of UCNPs was varied in the experiment. With a low concentration of UCNPs (0.2 mg ml^−1^) a large number of SiO_2_ NPs were obtained (see Fig. 2A). However, as the concentration of UCNPs was progressively increased to 1.3 mg ml^−1^, the number of SiO_2_ NPs was gradually reduced (see Fig. 2B and C) until none were observed (see Fig. 2D). The silica growth is evident from the appearance of a lower contrast layer surrounding the inorganic nanoparticle core in the acquired TEM images. It is also elucidated by FTIR characterization with the appearance of a stretching vibration attributed to Si–O–Si bonds (Fig. S5†).

The experimental conditions determined above for sample 1 cannot be adapted to other types of nanoparticles, accordingly. For larger UCNPs of equal mass, the number of nanoparticles will vary and will not match the number of aqueous micelles. This will also apply for UCNPs of varying morphology. We therefore devised a general approach to silica coating where the surface area of UCNPs was used to determine the necessary concentration of UCNPs to avoid the formation of SiO_2_ NPs.

### Determination of the total surface area (SA_T_) of UCNPs

A general approach should take into account not only the size, but also the shape of the UCNPs (spherical, hexagonal, rod-like, *etc.*) because the surface area is different for particles of different morphologies. For this reason, the SA_T_ is the most relevant parameter which correlates with the appropriate concentration of UCNPs. Initially, the SA_T_ of the as-prepared UCNPs (sample 1), was calculated.

Due to their near spherical shape the following equation was used to obtain the total surface area of a sphere, where *r* is the radius of the spherical nanoparticle:(SA)_sphere_ = 4π*r*^2^

It was determined that the UCNPs had a corresponding surface area of 1596 nm^2^ and this was correlated to the nanoparticle concentration (1.3 mg ml^−1^), ideal to obtain a high yield of silica-coated UCNPs. Accordingly, the SA_T_ values of sample 2–5 were calculated taking into account the general formula for right hexagonal prisms (see Scheme S2†):

1 (SA)_prism_ = 2*A* + *M*

2
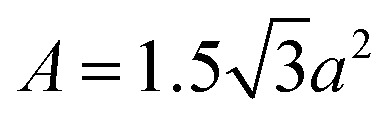

3 *M* = *ha*6

where *A* and *M* indicate the surface area of the base and lateral prism and *a* and *h* are the prism side length and the height, respectively.

Following determination of the SA_T_ values, the theoretical concentration of UCNPs could be estimated by assuming a linear relationship with sample 1. Table 1 shows the estimated concentrations for samples (2–5), along with their SA_T_, required to satisfy the linear relationship in order to avoid the formation of SiO_2_ NPs.

**Table 1:** SA_T_ values and concentration of NaYF_4_: Yb^3+^ (20%), Er^3+^ (2%) UCNPs for samples (2–4)

Sample used as core	Total surface area SA_T_ (nm^2^)	UCNPs (mg ml^−1^)
Sample 2	6281	5.1
Sample 3	3185	2.6
Sample 4	2101	1.7
Sample 5	20 850	17

### Silica coating of UCNPs with various morphologies

**Fig. 3:**
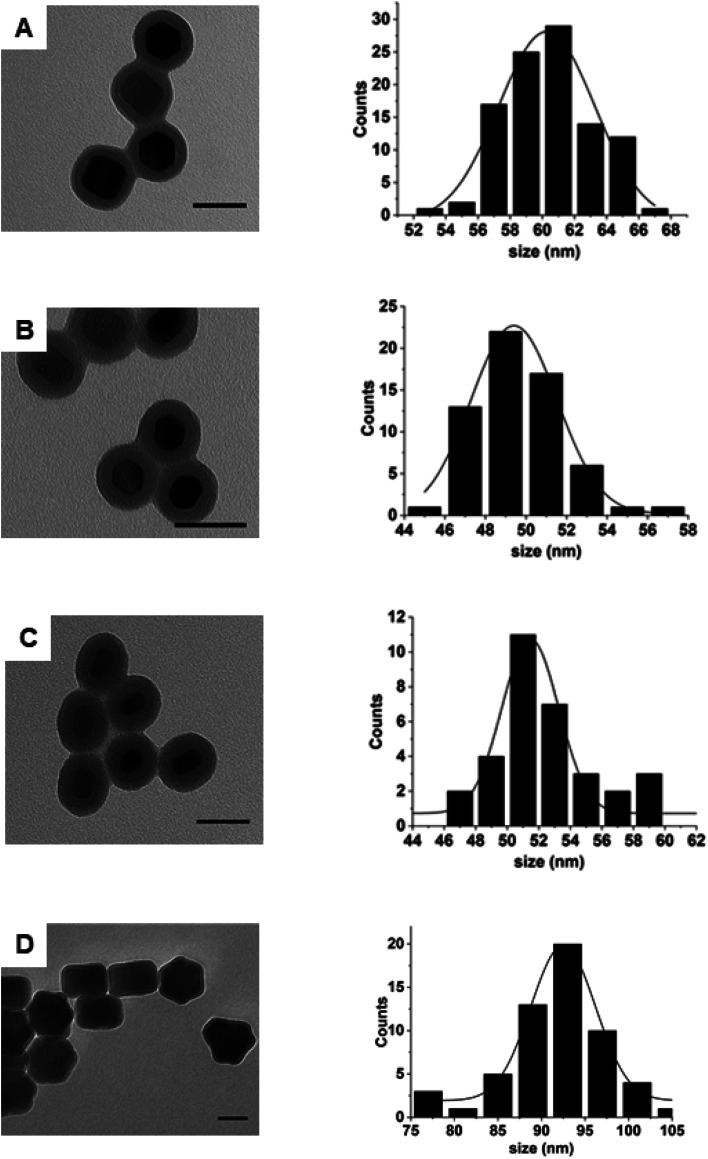
TEM images and size distribution of silica-coated UCNPs using sample 2 (A), sample 3 (B), sample 4 (C) and sample 5 (D) as a nanoparticle template. Representative histograms indicate the diameter of the silica-coated UCNPs. Scale bar is 50 nm.

Following estimation of the necessary theoretical concentrations needed for silica growth on the surface of nanoparticles, we proceeded to determine whether it could be experimentally applied to nanoparticles of samples 2–5. Fig. 3 shows that following silica growth, the UCNPs were monodisperse in size and no SiO_2_ NPs were obtained as a side product (see Fig. S6† for low magnification images). The silica shells appeared uniform in thickness and regular in surface. The silica-UCNPs size for each sample was measured to be 61 nm ± 2 nm, 60 nm ± 3 nm, 49 nm ± 2 nm and 93 ± 4 for sample 2–5, respectively. In each case we noticed that although the TEOS amount was kept the same for all samples, the thickness of the silica shells decreased as the surface area of the UCNPs increased as shown in Table S2.† The thickness of the silica shell was estimated from the difference between the mean diameters of UCNPs coated with silica and the UCNPs cores.

### Preventing nanoparticle aggregation during silica shell growth

Formation of aggregates of nanoparticles coexisting under the same silica shell is another major drawback that arises following silica growth on UCNPs. For practical applications, it is imperative that every nanoparticle is coated with silica homogenously and individually and nanoparticle dispersibility plays a critical role here. We observed that occasionally in our experiments UCNPs easily agglomerated and bundled together prior to silica coating, which during the growth reaction produced silica-coated nanoparticle aggregates (see Fig. S7†). Agglomeration of nanoparticles resulted from loss of OA surface ligands caused during purification. Indeed, it has been reported, that the OA can easily dissociate from the nanoparticle surface by overtreating the UCNPs with an excess of ethanol under sonication.^33^ Bian and co-workers reported a synthesis to improve the crystallinity of α-UCNPs, reconstructing the crystal edges of nanoparticles following a wet chemical and thermal annealing in the presence of OA.^30^ We speculated that this treatment could also help to improve the colloidal dispersibility of our UCNPs.

**Fig. 4:**
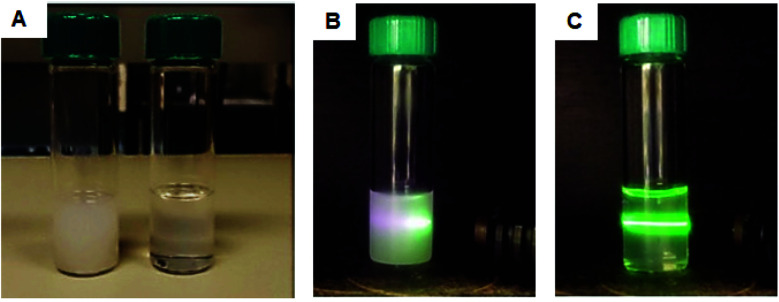
(A) Digital images of UCNPs prior (left) and post (right) annealing dispersed in hexane under ambient light. Laser irradiation of UCNPs before (B) and after (C) annealing.

UCNPs were annealed at high temperature in the presence of excess OA and compared to the non-annealed UCNPs at the same concentration and same conditions. Fig. 4 shows that post-treatment with OA increased the colloidal stability of the UCNPs in organic solvents. Prior to annealing, a turbid solution of UCNPs was observed. This was due to scattering of nanoparticle agglomerates deriving from the poor nanoparticle colloidal dispersibility. The annealing treatment resulted in a change in the transparency of the solution from turbid to clear indicating the formation of colloidally stable and well-disperse UCNPs. These well dispersed nanoparticles corresponded to sample 1, and they were used for the successful silica growth on the nanoparticle surface (see Fig. 2).

## Conclusion

Our work provides an insight into the steps that need to be followed to achieve high yield homogeneous silanization of UCNPs regardless of their shape and size. It was shown that by calculating the surface of UCNPs, the optimum concentration of UCNPs (mg ml^−1^) required during silica growth could be determined. For each type of nanoparticle a homogeneous silica coating was achieved whilst avoiding the formation of SiO_2_ NPs. Furthermore, we demonstrated how agglomeration of silica-coated UCNPs can be avoided by performing an annealing treatment of nanoparticles to ensure their colloidal dispersibility prior to the silica growth. Our step-by-step approach can be applied to OA-coated inorganic nanoparticles of varying shapes and sizes, overcoming the limitations that hinder their successful silanization and thus enable their broader applicability.

## Experimental section

### Materials

All the reagents for the synthesis of UCNPs, were purchased form Sigma-Aldrich. These are: lanthanide chloride (99%): yttrium(iii) chloride hexahydrate, ytterbium(iii) chloride hexahydrate, erbium(iii) chloride hexahydrate, thulium(iii) chloride hexahydrate, holmium(iii) chloride hexahydrate, gadolinium(iii) chloride hexahydrate, lutetium(iii) chloride hexahydrate, oleic acid (OA, 90%), 1-octadecene (ODE, 90%), sodium hydroxide (NaOH, ≥ 98%), ammonium fluoride (NH_4_F, ≥ 98%), polyoxyethylene (5) nonylphenylether (IGEPAL CO-520, average Mn 441), tetraethyl orthosilicate (TEOS, ≥99%), and (3-aminopropyl) trimethoxysilane (APTMS, 97%).

### Synthesis of NaYF_4_: Yb^3+^ (20%), Er^3+^ (2%) nanoparticles – sample 1

NaYF_4_: Yb^3+^ (20%), Er^3+^ (2%) nanoparticles were synthesized following a previously reported protocol with slight modifications.^28^ The synthesis was done using a Schlenk-line. In a 100 ml three-neck round-bottomed flask, YCl_3_·6H_2_O (0.78 mmol, 236 mg), YbCl_3_·6H_2_O (0.20 mmol, 77.50 mg), and ErCl_3_·6H_2_O (0.02 mmol, 7.63 mg) were mixed together with 6 ml of OA (90%) and 15 ml of ODE (90%). Then, the solution was heated to 150 °C under the presence of Ar gas for 1 h and 30 min. After that, the solution was cooled down to room temperature, and a mixture of NaOH (2.5 mmol, 100 mg) and NH_4_F (4 mmol, 148.16 mg) dissolved in 10 ml of anhydrous methanol was added dropwise under vigorous stirring. The mixture was stirred for 45 min at room temperature. After evaporating the methanol under Ar by heating to 100 °C for 30 min, vacuum was applied for a further 30 min at 100 °C. Finally, the solution was heated up to 300 °C and maintained for 1 h and 10 min under an Ar atmosphere. The solution was cooled to room temperature, and the UCNPs were precipitated with ethanol and washed by centrifugation three times (8000 rpm, 15 min). Each time the pellet was dispersed in ethanol. The as-prepared nanoparticles were dried and weighted. Then, they were re-dispersed in hexane and stored at room temperature for further use.

### Synthesis of NaYF_4_: Yb^3+^ (20%), Er^3+^ (2%) nanoparticles with different morphology – sample 2–4

Samples (2–4) consisting of NaYF_4_: Yb^3+^ (20%), Er^3+^ (2%) nanoparticles were synthesized by a slightly modified procedure to the experimental protocol used for the synthesis of sample 1. Here, the OA volume was gradually increased in the reaction mixture as following: 12 ml, 17 ml and 21 ml while the ODE volume was kept the same for all experiments (15 ml). Furthermore, during the last heating step the reaction temperature and the reaction time were set at 305 °C and 1 h and 20 min, respectively.

### Synthesis of NaY (28%)/Lu (50%) F_4_: Yb^3+^ (20%), Er^3+^ (2%) nanoparticles-sample 5

The synthesis of β-NaY (28%)/Lu (50%) F_4_: Yb^3+^ (20%), Er^3+^ (2%) nanoparticles was performed as previously described with slight modifications.^29^ In detail, 1 mmol of RECl_3_·6H_2_O was mixed together with 6 ml of OA and 15 ml of ODE in a 100 ml three-neck round-bottomed flask. The concentrations were YCl_3_·6H_2_O (0.58 mmol, 175.95 mg), YbCl_3_·6H_2_O (0.20 mmol, 77.5 mg), ErCl_3_·6H_2_O (0.02 mmol, 7.63 mg) and LuCl_3_·6H_2_O (0.50 mmol, 194.71 mg). The solution was heated to 150 °C under the presence of Ar gas for 1 h and 30 min to achieve a complete solubilisation of salts. After that, the solution was cooled down to room temperature, and a mixture of NaOH (2.5 mmol, 100 mg) and NH_4_F (4 mmol, 148.16 mg) in 10 ml of methanol was added. The mixture was stirred for 45 min at room temperature and after evaporating the methanol under Ar by heating to 100 °C for 30 min, vacuum was applied for 30 min at 100 °C. Finally, the solution was heated up to 308 °C and maintained for 1 h and 35 min under an Ar atmosphere. The solution was cooled down naturally and the UCNPs were precipitated with ethanol and washed by centrifugation three times (8000 rpm, 15 min). Each time the pellet was dispersed in ethanol. The as-prepared nanoparticles were dried and weighted. Then, they were re-dispersed in hexane and stored at room temperature for further use.

### Annealing treatment of upconversion nanoparticles

The UCNPs were annealed, if agglomerated, following a reported protocol with minor modifications.^30^ In detail, OA coated upconversion nanoparticles (100 mg) in hexane (10 ml) were added to a 100 ml three-neck round bottom flask together with OA (8 ml) and ODE (12 ml). The mixed solution was heated up gradually to 100 °C to evaporate the hexane. After 40 min, the temperature was increased to 240 °C and maintained for 1 hour and 30 min under Ar gas. The resulting annealed nanoparticles were cooled down naturally, collected by centrifugation and washed with ethanol three times (8000 rpm, 15 min). The annealed β-UCNPs were re-dispersed in hexane and stored for further use.

### Growth of silica on the upconversion nanoparticles

A solution containing an appropriate amount of the nanoparticles dispersed in 10 ml of hexane was mixed with Igepal Co-520 (0.5 g, average *M*_n_ 441) and sonicated for 15 min. Ammonium hydroxide (NH_4_OH) (35%, 100 μl) was then added and the solution was shaken and sonicated vigorously for 20 min. Finally, tetraethyl orthosilicate (TEOS) (75 μl) was injected into the solution under continuous stirring. The mixture was left stirring overnight and the precipitate was collected by centrifugation (8000 rpm, 10 min) and washed with ethanol three times. The resulting nanoparticles coated with silica (β-UCNPs@SiO_2_) were re-dispersed in 5 ml ethanol and stored for further use.

### Characterization

Transmission electron microscopy (TEM) was performed on a Hitachi HT7700 operating at 80 kV. The nanoparticle size distribution was analysed with ImageJ (National Institutes of Health, USA) software considering over 150 nanoparticles for the statistical analysis. XRD analysis was performed using a Bruker D2 phaser X-ray diffractometer equipped with a CuKα source (*λ* = 1.54059 nm). Fourier transform infrared (FT-IR) spectra were recorded in the spectral range of 525 and 4000 cm^−1^ on a Nicolet iS5 FT-IR spectrometer at room temperature. For the sample preparation, nanoparticles were dried and deposited directly on the glass (approximately 1 mg required for analysis).

## Conflicts of interest

The authors declare no competing financial interest.

## Supplementary Material

NA-003-D0NA00858C-s001
