# Supplementary material for: A method for the growth of uniform silica shells on different size and morphology upconversion nanoparticles
Source: Nanoscale Adv. 2021 May 10;3(12):3522–9. doi: 10.1039/d0na00858c (PMC8204744; doi:10.1039/d0na00858c)
Supplement: NA-003-D0NA00858C-s001 [file NA-003-D0NA00858C-s001.pdf]

Electronic Supplementary Material (ESI) for Nanoscale Advances.
This journal is © The Royal Society of Chemistry 2021

Supplementary Information

A Method for the Growth of Uniform Silica Shells on Different Size and Morphology Upconversion Nanoparticles

Elena Ureña-Horno,[a] Maria-Eleni Kyriazi,[a] and Antonios G. Kanaras[a,b,*]

[a] Physics and Astronomy, Faculty of Physical Sciences and Engineering, University of Southampton, Southampton SO171BJ, United Kingdom.

[b] Institute for Life Science, University of Southampton, Southampton, SO171BJ, United Kingdom.

Table of Contents

S-I Characterization of upconversion nanoparticles (UCNPs)

a. **Size histogram of $NaYF_4$: Yb^{3+} (20%), Er^{3+} (2%) UCNPs – Sample 1**
b. **Dimensions of UCNPs and corresponding histograms**
c. **X-Ray diffraction (XRD) of UCNPs**
d. **Fourier-transform infrared (FT-IR) characterization**

S-II Characterization of silica-coated UCNPs

a. **FT-IR characterization**
b. **Surface area of hexagonal prism**
c. **Supplementary TEM images**
d. **Silica shell thickness**

S-III Characterization of annealed UCNPs

a. **TEM images of silica-coated UCNPs without annealing treatment**
b. **TEM images of silica-coated UCNPs after annealing treatment**

S-I Mechanism of silanization *via* reverse microemulsion

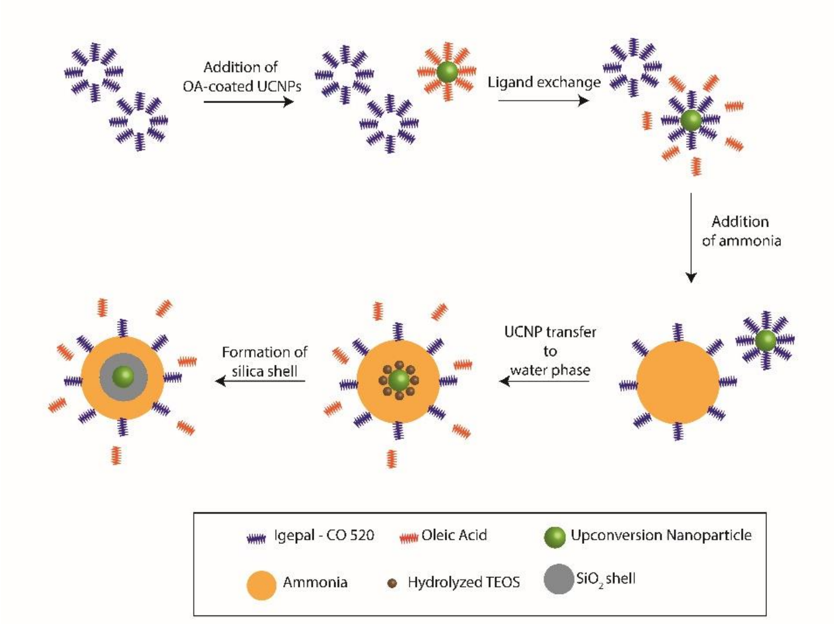

Scheme S1. Schematic illustration of the formation of a silica shell around UCNPs *via* the reverse microemulsion system. Briefly, Igepal CO-520 forms micelles and replaces the oleic acid on the surface of UCNPs. Upon the addition of ammonia, the micelles are enlarged. TEOS is then added, which is hydrolysed and replaces the Igepal CO-520 on the surface of UCNPs facilitating their transfer into the water phase. Finally, a silica shell is then formed around the UCNPs.[1]

S-II Characterization of UCNPs

a. Size histogram of $NaYF_4$: Yb^{3+}(20%), Er^{3+}(2%) UCNPs-sample 1

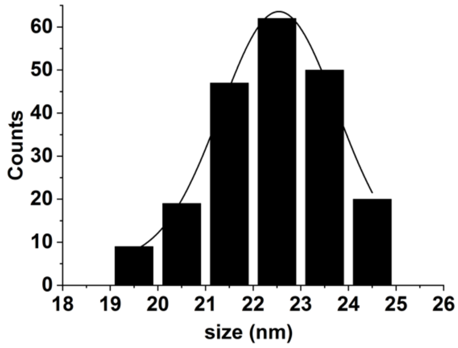

Figure S1. Size distribution of $NaYF_4$: Yb^{3+}(20%), Er^{3+}(2%) UCNPs analysed using ImageJ. Particles appeared uniform in morphology with an average size of 23 nm and a standard error of 1 nm.

b. Dimensions of UCNPs and corresponding histograms

Table S1. Dimensions of $NaYF_4$: Yb^{3+}(20%), Er^{3+}(2%) and NaY(28%)/Lu(50%)F_4: Yb^{3+}(20%), Er^{3+}(2%) UCNPs obtained by using increasing amounts of OA

	Shape	Core diameter (nm)	Length (nm)	Aspect ratio
Sample 1	Spherical	23 ± 1	-	-
Sample 2	Hexagonal	39 ± 2	37 ± 3	0.94
Sample 3	Rod	25 ± 2	32 ± 2	1.28
Sample 4	Rod	19 ± 1	28 ± 1	1.47
Sample 5	Hexagonal	83 ± 4	52 ± 3	0.63

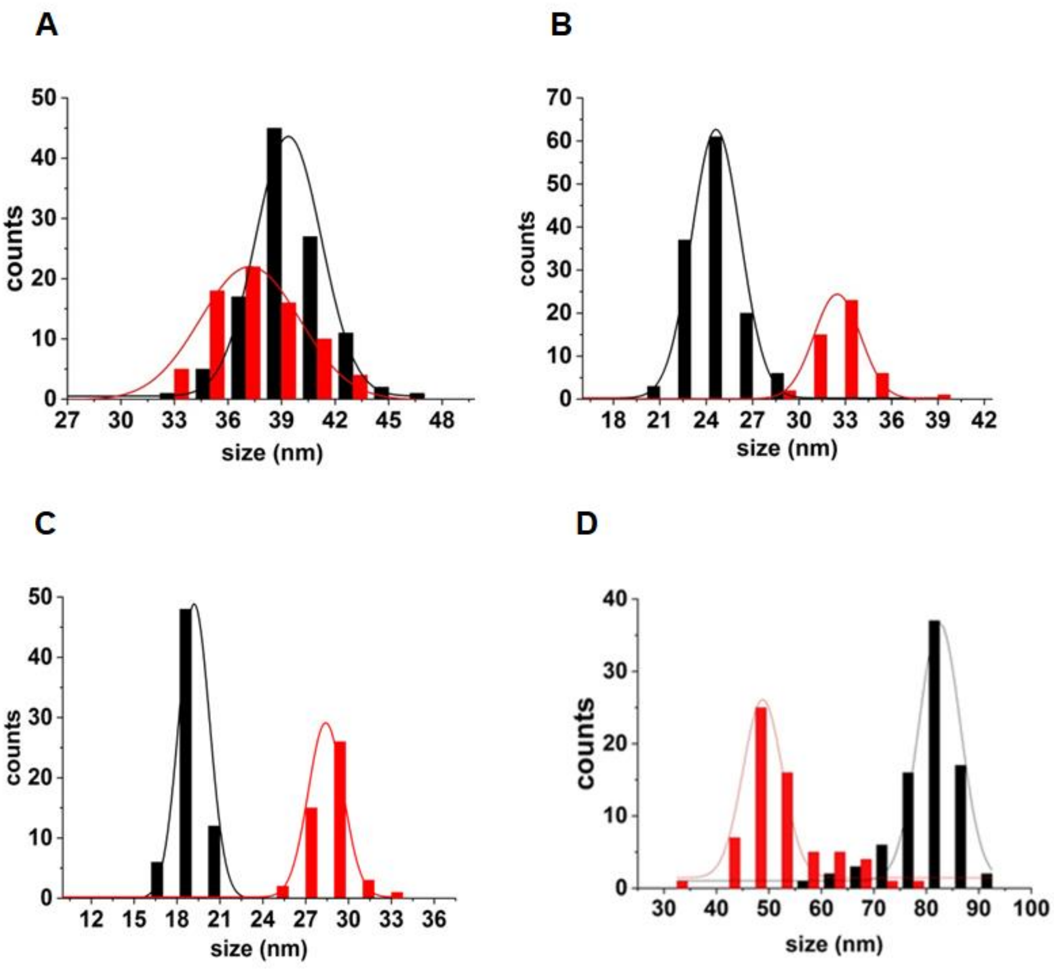

Figure S2. Size distribution of $NaYF_4$: Yb^{3+}(20%), Er^{3+}(2%) UCNPs synthesised using (A) 12, (B) 17 and (C) 21 mL of OA as well as large hexagonal NaY (28%)/Lu(50%)F_4: Yb^{3+}(20%), Er^{3+}(2%) UCNPs (D). Black bars represent the core diameter whereas the red bars indicate length of UCNPs. Nanoparticle dimensions were analysed using ImageJ.

c. X-Ray diffraction (XRD) of UCNPs

The crystal structure of the synthesised UCNPs was assessed *via* XRD. When compared to the standard pattern for the hexagonal phase $NaYF_4$: Yb; Er nanoparticles it could be deduced that UCNPs displayed a hexagonal crystalline structure.

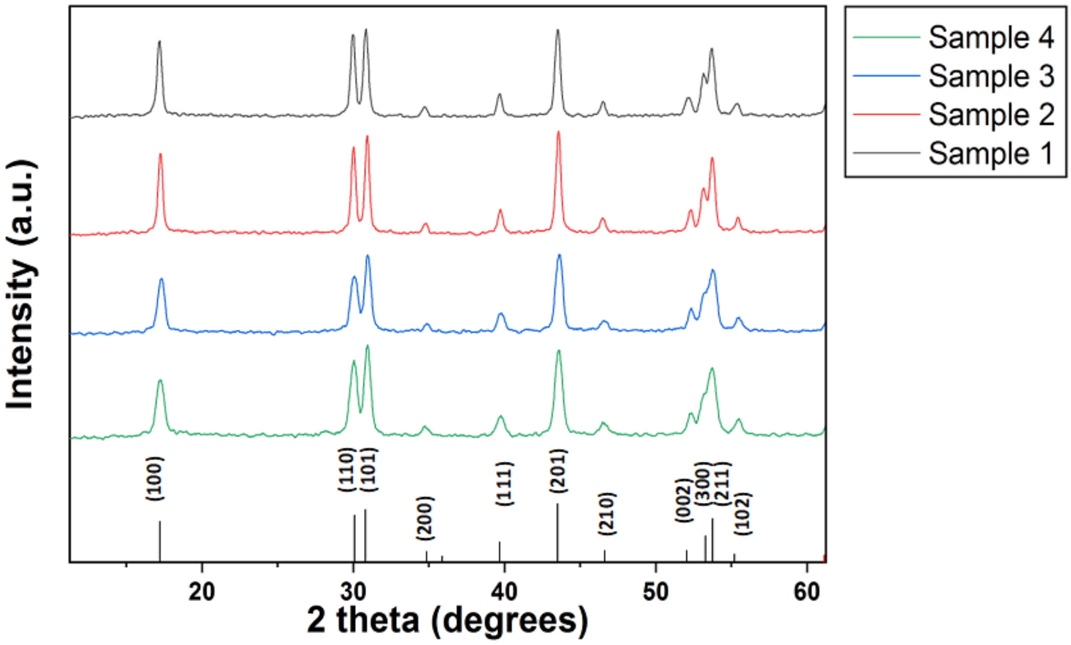

Figure S3. XRD patterns of $NaYF_4$: Yb^{3+}(20%), Er^{3+}(2%) UCNPs prepared with different OA concentrations confirming the hexagonal crystallinity of the host lattice. Measurements were acquired using a Cu K-α source (with λ = 1.54059 Å). The vertical lines on the x axis correspond to the standard pattern for the hexagonal phase $NaYF_4$: Yb; Er nanoparticles (PDF card No.: 00-016-0334).

d. Fourier-transform infrared (FT-IR) characterization

The OA coating on the surface of the UCNPs was further confirmed by FT-IR spectroscopy as shown in **Figure S4**. Peaks at 2923, 2858, 1559, 1459 and 1110 cm^{-1} are characteristic of the OA molecule confirming the ligand coating.

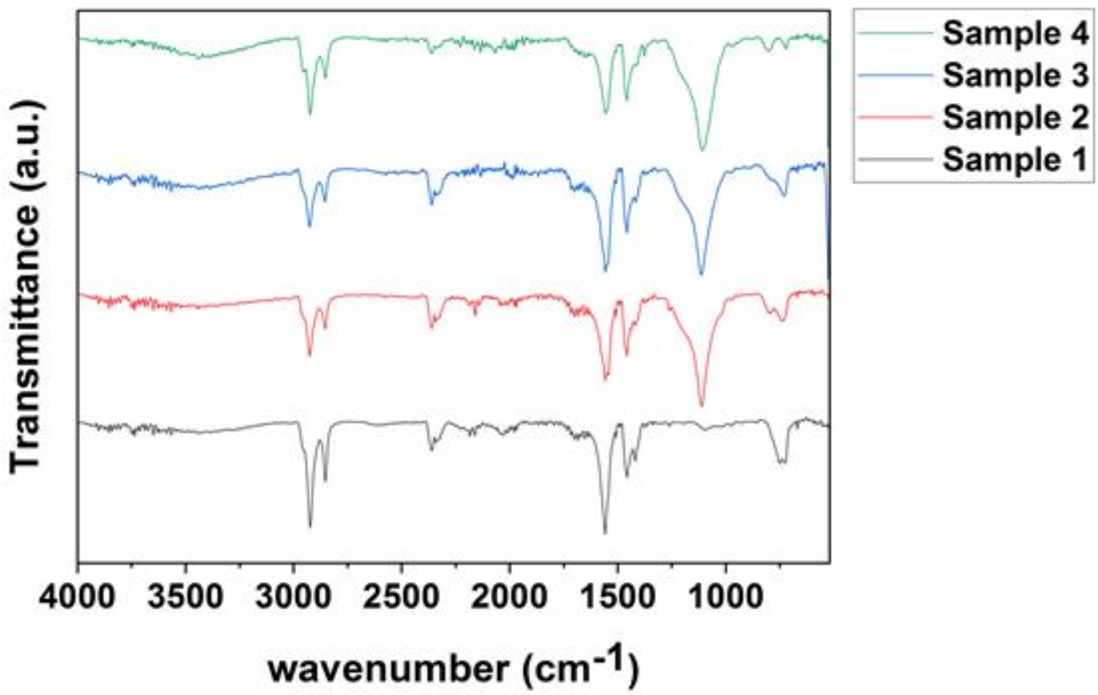

Figure S4. FT-IR measurements of OA-capped UCNPs synthesised with increasing volumes of OA. Peaks at 2923 and 2858 cm^{-1} were attributed to the symmetric and asymmetric stretching modes of the methylene group. Bands at 1559 and 1459 cm^{-1} were attributed to the asymmetric and symmetric stretching vibrations of the carboxylate group [$(–COO–)_3Y^{3+}$] of the β-$NaYF_4$, respectively whereas the absorption band at 1110 cm^{-1} was due to the C-O bond.

S-III Characterization of silica-coated UCNPs

a. FT-IR characterization

Apart from transmission electron microscopy, successful silica coating of UCNPs was also verified by FT-IR spectroscopy. **Figure S5** shows that characteristic peaks that are attributed to the OA coating were no longer present. Compared to the FT-IR spectrum of UCNPs prior to silanization only one main peak appeared at 1109 cm^{-1}, which was attributed to the Si-O-Si stretching vibrations.

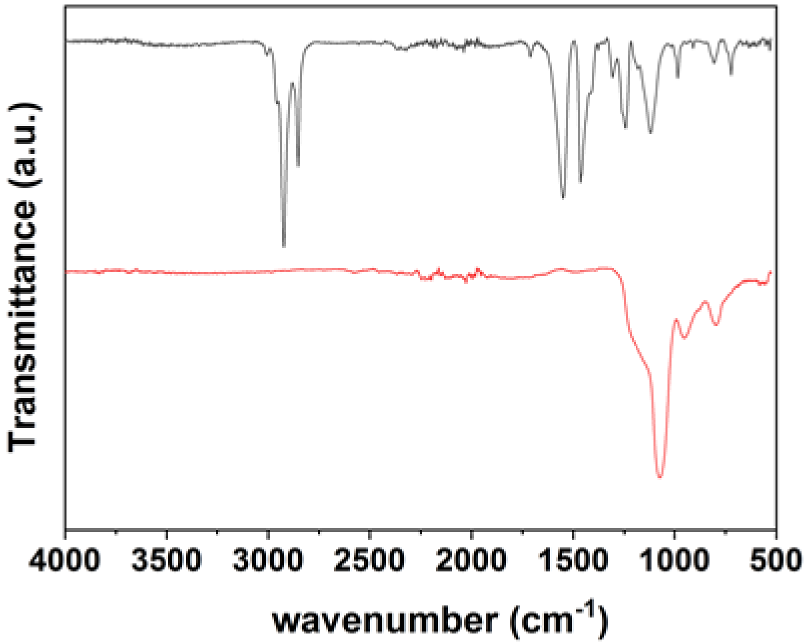

Figure S5. FT-IR spectra of spherical $NaYF_4$: Yb^{3+}(20%), Er^{3+}(2%) UCNPs before (black line) and after silica coating (red line).

b. Surface area of a hexagonal prism

The surface area of the hexagonal UCNPs was determined according to the formula which takes into account the base edge and height of UCNPs. **Scheme S2** shows an illustration of a hexagonal prism where both parameters have been annotated.

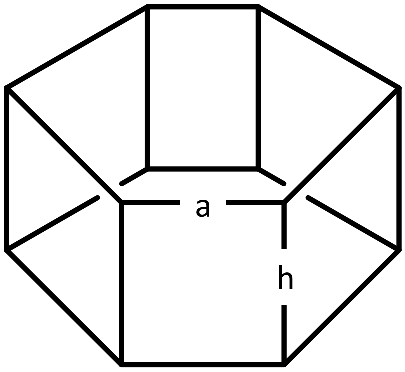

Scheme S2. Schematic illustration of a hexagonal prism. The surface area of hexagonal UCNPs was calculated by taking into account their base edge (a) and height (h).

c. **Supplementary TEM images**

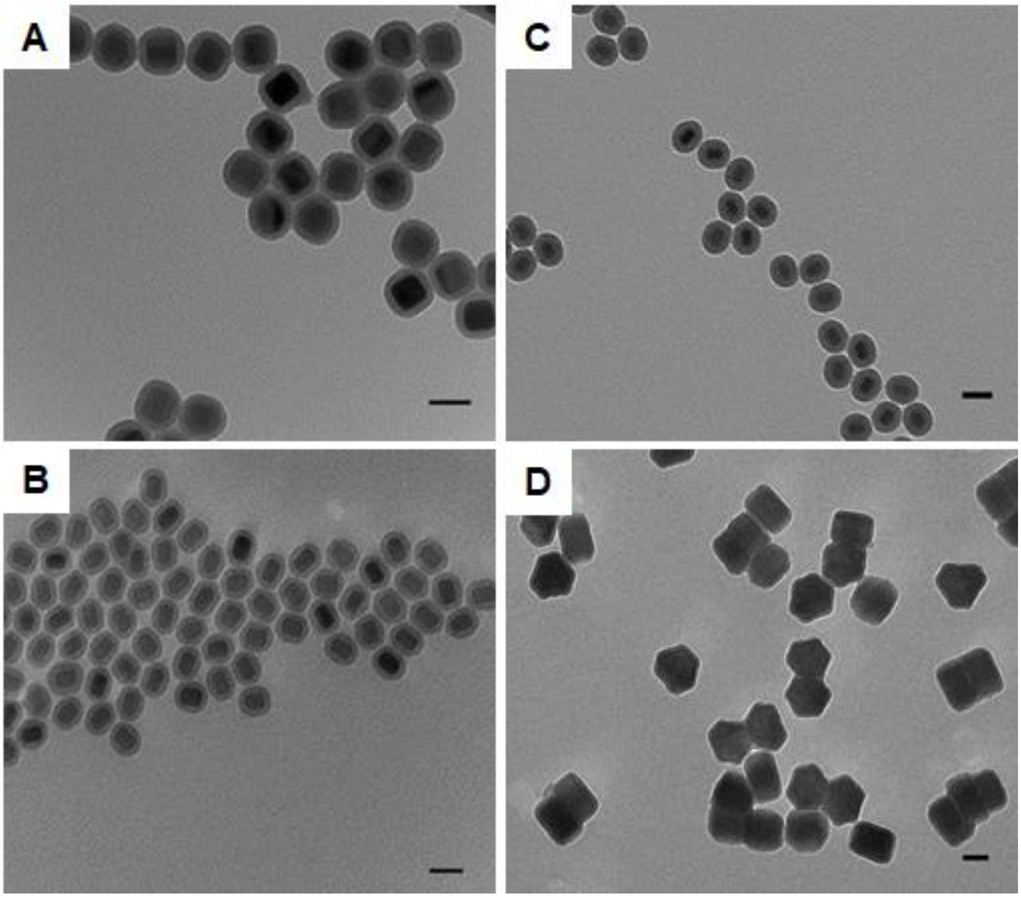

Figure S6. Supplementary TEM images of sample 2 (A), sample 3 (B), sample 4 (C) and sample 5 (D) showing that no empty silica shells are formed following silanization. Scale bar is 50 nm.

d. Silica shell thickness

Table S2. Calculated silica shell thickness for UCNPs of varying size and morphology. With increasing surface area the silica shell thickness decreases.

	Core diameter (nm)	SA_T (nm^2)	UCNPs (mg/ml)	Silica thickness (nm)
$NaYF_4$: Yb^{3+}(20%), Er^{3+}(2%)	39 ± 2	6281	5.1	~11
$NaYF_4$: Yb^{3+}(20%), Er^{3+}(2%)	25 ± 2	3185	2.6	~14
$NaYF_4$: Yb^{3+}(20%), Er^{3+}(2%)	19 ± 1	2101	1.7	~17
NaY(28%)/Lu(50%)F_4: Yb^{3+}(20%), Er^{3+}(2%)	83 ± 4	20850	17.0	~5

S-IV Characterization of annealed UCNPs

c. TEM images of silica-coated UCNPs without annealing treatment

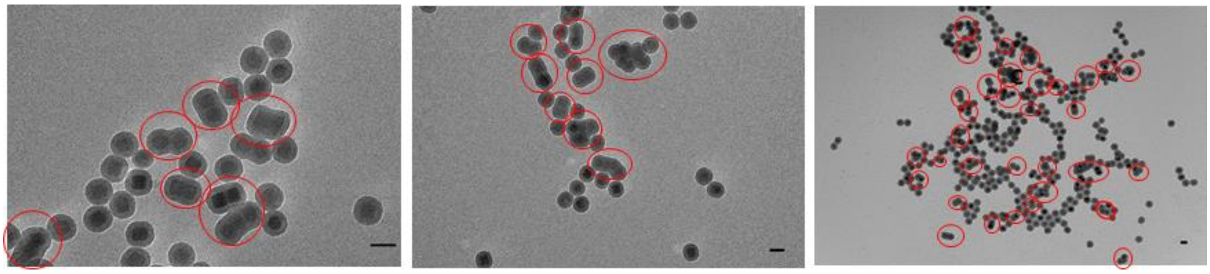

Figure S7. TEM images of silica-coated UCNPs. Red circles indicate silica-coated nanoparticle agglomerates that occur due to poor colloidal dispersibility of the initial UCNPs. Scale bars are 50 nm.

REFERENCES

1. Ding, H. L.; Zhang, Y. X.; Wang, S.; Xu, J. M.; Xu, S. C.; Li, G. H., Fe3O4@SiO2 Core/Shell Nanoparticles: The Silica Coating Regulations with a Single Core for Different Core Sizes and Shell Thicknesses. *Chem. Mater.*, 2012**, 24**, 4572-4580.
